# Uncovering Differentially Methylated Regions (DMRs) in a Salt-Tolerant Rice Variety under Stress: One Step towards New Regulatory Regions for Enhanced Salt Tolerance

**DOI:** 10.3390/epigenomes3010004

**Published:** 2019-01-18

**Authors:** Liliana J. Ferreira, Mark T. A. Donoghue, Pedro Barros, Nelson J. Saibo, Ana Paula Santos, M. Margarida Oliveira

**Affiliations:** 1 Instituto de Tecnologia Química e Biológica António Xavier, Universidade Nova de Lisboa, Genomics of Plant Stress. Av. da República, 2780-157 Oeiras, Portugal; 2 Cold Spring Harbor Laboratory, Cold Spring Harbor, NY 11724, USA; 3 IBET, Apartado 12, 2781-901 Oeiras, Portugal

**Keywords:** differentially methylated regions (DMRs), MeDIP-Seq, rice, salt stress tolerance

## Abstract

Chromatin structure, DNA methylation, and histone modifications act in a concerted manner to influence gene expression and therefore plant phenotypes. Environmental stresses are often associated with extensive chromatin rearrangements and modifications of epigenetic levels and patterns. Stress-tolerant plants can be a good tool to unveil potential connections between specific epigenetic modifications and stress tolerance capacity. We analyzed genome wide DNA methylation of a salt-tolerant rice variety under salinity and identified a set of differentially methylated regions (DMRs) between control and stress samples using high-throughput sequencing of DNA immunoprecipitated with the 5-methylcytosine antibody (MeDIP-Seq). The examination of DNA methylation pattern at DMRs regions revealed a general tendency for demethylation events in stress samples as compared to control. In addition, DMRs appear to influence the expression of genes located in their vicinity. We hypothesize that short regions as DMRs can shape the chromatin landscape of specific genomic regions and, therefore, may modulate the function of several genes. In this sense, the identification of DMRs represents one step towards to uncover new players in the regulation of stress-responsive genes and new target genes with potential application in enhancement of plant salinity-tolerance.

## 1. Introduction

Rice is well known for its extreme sensitivity to salinity which may lead to reduced levels of productivity and negative impacts on growth rates, tillering and seed production [1,2,3]. Thus, distinct perspectives are needed to enhance knowledge about rice tolerance and adaptation to adverse environmental conditions. Several abiotic stresses have been studied such as suboptimal temperature, water and nutrient availability, light, salinity, and temperature conditions. The enhancement of salt tolerance has been achieved by the production of transgenic plants [4]. For example, in rice the overexpression of specific genes, such as *OsSta2-D* (*Oryza sativa* Salt tolerance activation 2-Dominant), generated positive impacts on better rice performance under salt stress [5]. Similarly, the overexpression of dehydrin gene, *OsDhn1*, led to enhanced performance of rice plants subjected to drought and salt since these plants showed a high capacity to minimize the level of reactive oxygen species (ROS) in cells increasing their tolerance to imposed oxidative stress [6]. Other approaches to uncover the molecular mechanisms underlying abiotic stress responses have involved the identification of miRNA profiles [7], for example, in maize, the downregulation of specific miRNA was detected in response to salt stress [8]. Genome wide demethylation and structural chromatin remodeling are also common events resulting from exposure to salt stress [9,10,11,12,13]. In *Antirrhinum majus*, the exposition to low temperatures induced DNA demethylation of Tam3 sequence which was correlated to its activation [14]. The germination of rice seeds under salt stress conditions also generated a global decrease of DNA methylation [13] and a remarkable decondensation of interphase rDNA chromatin which became more evident at heterochromatic knobs [9]. The genome wide loss of DNA methylation of rice under salinity stress was found particularly evident in leaf tissues as compared to roots [11,12,13]. DNA methylation levels can be also altered in result of exposure to distinct chemical stresses. For example, toxicological studies in rice involving the application of atrazine (herbicide) revealed the existence of methylation changes at specific genes with a role in atrazine metabolism [15]. The exposure to chemical compounds targeting epigenetic regulators such as 5-azacytidine or trichostatin-A also affected the organization of interphase chromosomes and epigenetic levels in wheat nuclei [16]. Shifts in DNA methylation levels and patterns have been connected with plant capacities for adaptation and tolerance to stress. Rice genotypes with distinct degrees of susceptibility to various stresses possess distinct levels of DNA methylation and different abilities to adjust DNA methylation levels [13,17,18,19,20]. For instance, the salinity tolerant rice variety ‘Pokkali’ showed a higher ability to alter DNA methylation levels than sensitive varieties [13]. A salt-tolerant cultivar of foxtail millet (*Setaria italica*) under salinity stress also showed a genome wide loss of DNA methylation as compared to a sensitive one [21]. The ability to tolerate stress can also be influenced by genetic stress, namely by genome restructure after merge of distinct genomes in the same nucleus, as it happens in somatic hybridization or polyploidy [17,22], generating unpredictable reorganization of methylation patterns and novel maps of gene interactions [23]. For example, the enhanced salinity tolerance of salinity-tolerant wheat cultivar cv. SR3 was attributed to specific methylation changes arising from somatic hybridization [17]. Similarly, the drought-tolerance of rice line DK151 was attributed to extensive DNA methylation changes as result of the introgression feature of this line [19]. These studies indicated a connection between DNA methylation dynamics and plant capacity to tolerate stress.

The response to challenging situations has also been correlated with the induction of differential methylation regions (DMRs), being the DMRs location determinant in gene regulation [18,24,25]. DMRs have been mostly studied in humans and have been denominated according to their role specificity as tissue-specific DMRs (tDMR), cancer-specific DMRs (cDMR), reprogramming-specific DMRs (rDMR), imprinting-specific DMRs (iDMR), and aging-specific DMRs (aDMR) [26,27,28]. In plants, DMRs have been identified between different tissues along in vitro culture such as in Populus tissue dedifferentiation and regeneration [29]. DMRs were also identified between inbred lines of maize (*Zea mays*) [30], in soybean (*Glycine max*) [31], in rice hybrids [32], and also in Arabidopsis after an induced drought simulation [33]. DMRs were also identified between rice lines with contrasting behaviors in response to abiotic stresses. For example, rice drought-tolerant plants showed less drought-induced DMRs than drought-sensitive plants [19] which may suggest, as referred to by Zheng et al. [34], that drought-tolerance maybe associated to a more strength of methylome pattern under stress. 

Following our previous studies [13] we have furthered investigated the patterns of DNA methylation in ‘Pokkali’, a rice variety that simultaneously shows a great capacity to tolerate salinity stress and to rapidly shape DNA methylation levels when exposed to salinity. The methylome of ‘Pokkali’ was analyzed by methylated DNA immunoprecipitation (MEDIP-Seq) with focus on detection of DMRs upon salt stress exposure versus a control condition. The implementation of strict criteria for bioinformatics analysis led to the identification of 53 DMRs. Some of these regions were analyzed in detail by bisulfite sequencing (BS-Seq) and all identified DMRs revealed, in general, a loss of methylation upon salt. Moreover, most DMRs were found nearby specific genes and this may suggest that DMRs can be one more player involved in epigenetic gene regulation.

## 2. Results

### 2.1. The ‘Pokkali’ Methylome and the Identification of DMRs between Salinity and Control

**Figure 1:**
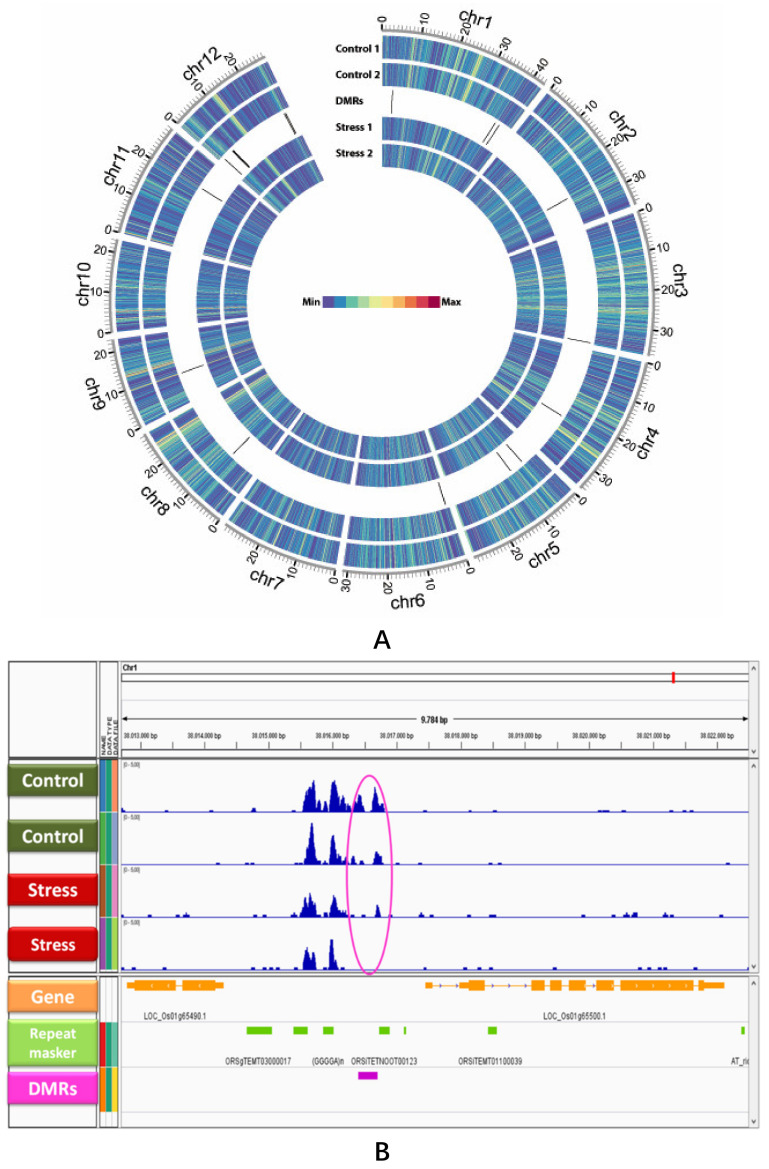
Identification of differentially methylated regions (DMRs) between control and salt stress samples in a salt-tolerant rice variety. (**A**) A genome-wide view of DNA methylation of Pokkali leaves. The circos plot representation was used to show the location of DMRs between control and salt stress conditions. The circos plot was based on the average RPM over 100,000 bp windows. (**B**) An example of a DMR (DMR 2) (pink bar and circle) exhibiting lower levels of DNA methylation in salt stress than in control conditions. The annotation of genes and repetitive sequences physically related to DMR is shown at the bottom. All the DMRs identified are shown in Figure S2.

The two sequencing runs performed generated 13.6 and 17.2 million 50 bp single end raw sequencing reads for salt and control conditions, respectively. After removal of adapter contaminants and low-quality reads, 3.5–4.2 million uniquely mapped high-quality reads were retained for each condition and replicate. The percentage of uniquely mapped reads was considerably higher on the non-immunoprecipitated sample (approximately 50%) contrasting with approximately 25% for the immunoprecipitated samples (Table 1). Regarding the genome coverage, approximately 7.5% of cytosines were covered by at least one uniquely mapped read (Table 1). The methylome of ‘Pokkali’ consisted on an even distribution of DNA methylation throughout the entire chromosomes, with no obvious enrichment on specific chromosome regions, such as pericentromeric heterochromatin (Figure 1A and Figure S1).

The analysis of differential methylation based on MeDIP-Seq data, using the MEDIPS program, as mentioned in the methods section, enabled the identification of 53 DMRs between control and salt stress samples. The DMRs that were close to each other (less than 500 bp) were merged originating 22 DMRs (ranging from 100 to 1000 bp) (Table 2). Regarding the DMRs profile, the methylation variation induced by salinity consisted, in general, of DNA demethylation (Figure 1B and Figure S2). For some DMRs, in control 2, there was also some loss of DNA methylation under stress (Figure S2) but the general tendency was a loss of methylation of DMRs under salinity stress. A detailed analysis of some DMRs by BS-Seq validated the loss of methylation upon salt stress (Figure S3). Although all methylation contexts were present (CG, CHG, and CHH), in DMR2 the methylation was mainly in the CHH context, while in DMR15 the CHG context was predominant. This analysis also revealed variation in methylated cytosines content among different DMRs. The DMR2 is considerably less methylated than DMR15 (2.012% and 5.51%, respectively) but both DMRs suffered a loss of methylation upon salt stress (1.066% and 4.494% for DMR2 and DMR15, respectively).

### 2.2. Location of DMRs Might Influence Regulation of Genes Nearby

**Figure 2:**
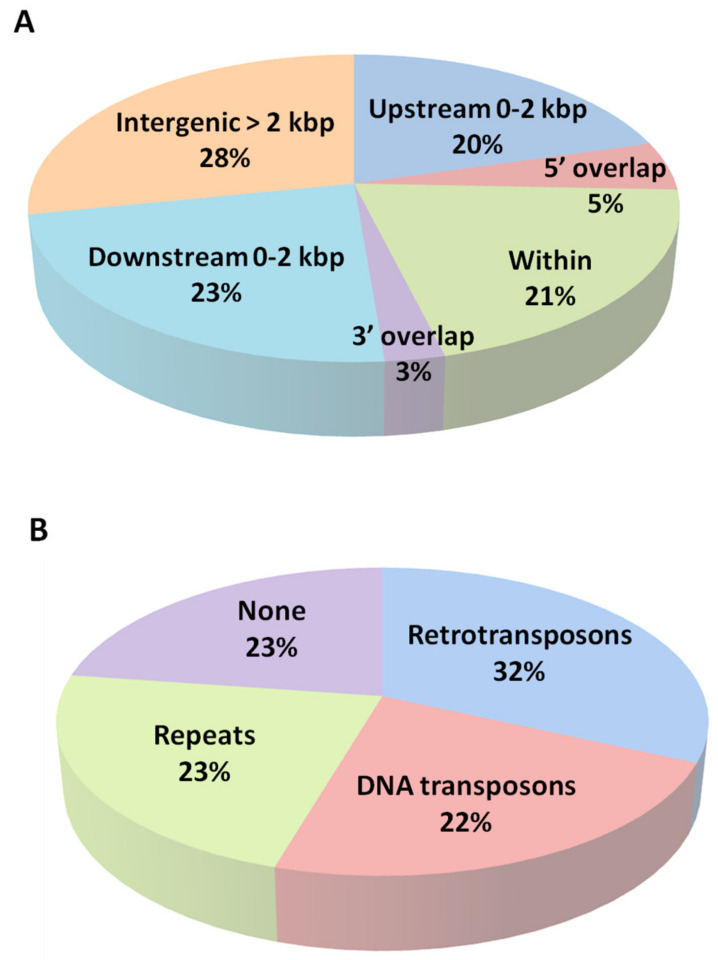
Classification of DMRs according to genomic features. (**A**) DMRs location in relation to their position to the nearest genes: Upstream [>2 kbp or between 0 and 2 kbp of the gene transcription starting site (TSS)], 5’ overlapping [in case the DMR overlaps to gene transcription starting site]; within [if the DMR falls completely within the borders of a gene]; 3’ overlapping, [in case the DMR overlaps with the 3’ end of an annotated gene]; or downstream [0 to 2 kbp or >2 kbp from gene end]. (**B**) DMRs location in relation to Repeat Masks annotation: Retrotransposons, Transposons, and Repeats [(CGG)n and (GGA)n rich areas].

The methylome is certainly shaped in response to stress but it is less clear how to connect changes on specific methylome patterns with modulation of gene expression. The DMRs location along rice chromosomes is shown in Table 2. A higher number of DMRs was detected in chromosomes VI and XII, four and six DMRS, respectively as shown in Table 2, while no DMRs were detected on chromosomes VII and X. Intersecting the MeDIP-Seq data with the rice genome annotation (http://rice.plantbiology.msu.edu/) and the Repeat Masker software (http://www.repeatmasker.org/), we could further obtain a genomic landscape for all DMRs (Figure S2). DMR2 is located on chromosome I, upstream a chloride channel protein coding gene (LOC_Os01g65500) and a DNA binding protein coding gene (LOC_Os01g65490), indicating that one DMR can potentially influence more than one gene (Figure 1B). The DMRs were analyzed according to their position relative to the nearest gene, and more than 70% of the DMRs were in close proximity to genes (less than 2 kbp away) (Figure 2A). Furthermore, over 75% of the DMRs identified were associated with transposable elements and repetitive sequences (Figure 2B).

**Figure 3:**
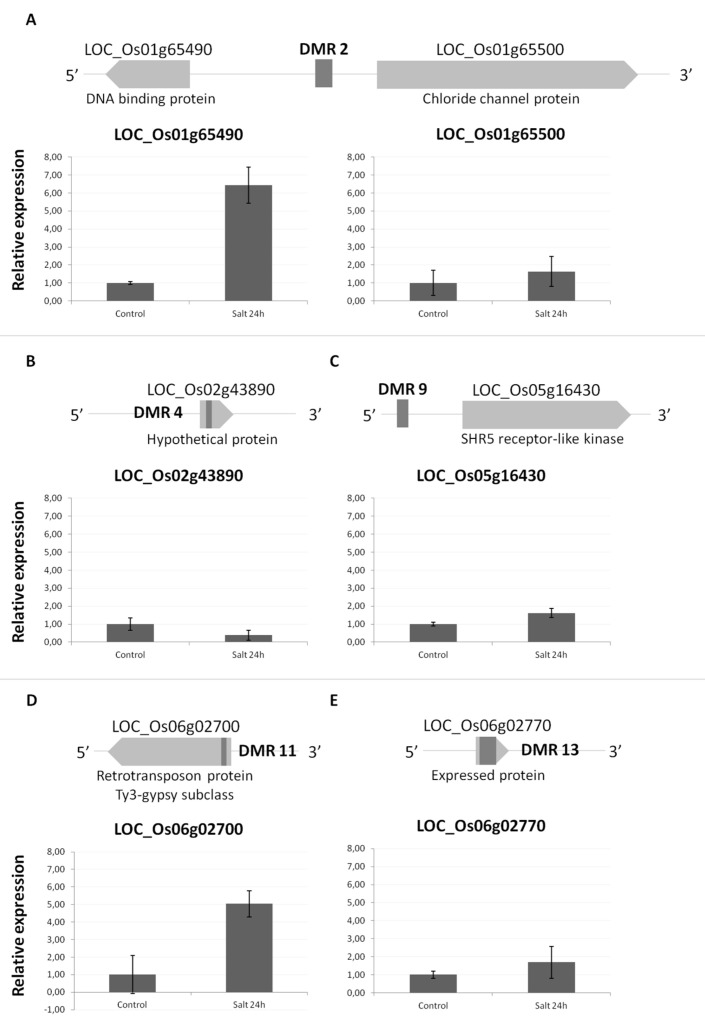
Expression studies of genes nearby DMRs by quantitative real-time qPCR. Genes containing DMRs at promoter regions are shown in (**A**) and (**C**) while genes containing DMRs within the coding region are shown in (**B**), (**D**), and (**E**). The mean expression value of control was normalized to 1 and the other mean values represent fold changes in expression of three technical replicates. The graphics show the result of one representative biological assay, from a total of three different replicates.

We further wanted to investigate the expression of several genes located nearby DMRs, before and after salt treatment by RT-qPCR. Based on the criterion of proximity to the identified DMRs, specific genes were selected for expression studies (Figure 3). The selected case studies included DMR2, located upstream two genes in opposite orientation (a DNA binding protein and a Chloride channel protein) (Figure 3A) and DMR9 (Figure 3C) located upstream a receptor-like kinase. Another three genes were selected for expression analyses because of carrying a DMR within the gene body (Figure 3B,D,E). The genes coding for a DNA binding protein and a retrotransposon (Figure 3A,D) showed a significant induction by salt stress, while a gene encoding a hypothetical protein (Figure 3B) was found to reduce expression upon stress. Regarding the other genes analyzed, no drastic changes were found upon stress.

The functional annotation of genes flanking or overlapping salt-induced DMRs was performed using Blast2GO [35] and multilevel pie charts were generated for the three main classes: cellular component, biological process, and molecular function (Figure S4). Regarding the molecular function, nine functional categories were identified, the most common being the protein binding followed by nucleotide binding and kinase activity (Figure S4A). For the cellular component, most of the proteins indicate plastidial location, but occasional location in the nucleus or in the plasma membrane was also identified (Figure S4B). The main biological processes annotated to those proteins are cellular protein modification processes, biosynthetic processes, carbohydrate, and DNA metabolic processes (Figure S4C).

## 3. Discussion

The study of epigenetic alterations in salt-tolerant plants can contribute to uncover the meaning of methylome changes in gene regulation and in stress tolerance. We previously reported that ‘Pokkali’, a salinity-tolerant rice variety, can display a quick relaxation of DNA methylation levels in response to salinity [13]. In this study, we wanted to deepen the methylome dynamics of ‘Pokkali’ and used the MeDIP-Seq approach to decipher differentially methylated regions (DMRs) between control and salt stress conditions. The MeDIP-Seq is a relatively affordable method to provide methylation information, although it does not allow an absolute quantification of the methylation nor is it sensitive enough to allow a high genome coverage [36,37]. In ‘Pokkali’, we found methylated areas mostly dispersed throughout all chromosomes with no clear evidences for high methylation density at specific chromosome regions as centromeres. Other studies in rice showed that centromeres are not densely methylated and even possess euchromatic subdomains at centromeric regions, compatible at same degree with gene transcription [38,39]. Furthermore, the rice interphase nuclei labeled with DAPI shows a diffuse chromatin organization pattern with no evidences of markedly labeled heterochromatic regions [40]. Contrastingly, DAPI staining applied to Arabidopsis nuclei easily enable the visualization of heterochromatic knobs [41] and the centromeric regions of Arabidopsis chromosomes were described as particularly rich in DNA methylation [42,43,44].

The investigation of genomic regions in leaf tissues of ‘Pokkali’ that could be preferentially selected for differential methylation in control and salt stress samples revealed a general tendency for DMRs to lose methylation upon salinity imposition which is in agreement with previous reports of demethylation associated with salt stress treatments [11,12,13]. By adopting strict filtering criteria in the bioinformatics analysis of the MeDIP-Seq data, 53 DMRs were identified either within genes or in its vicinity (less than 2kbp apart) and far from centromeres. A plausible hypothesis is that DMRs may modulate chromatin structure and in this way influence the transcriptional competence of specific genes depending on the DMRs location. A single DMR can be a regulation region influencing the expression level of several genes even if they locate physically apart [45]. The identification and location of DMRs, either in the exons, introns, or even in the exon/intron transition, may also bring new clues regarding the involvement of DNA methylation in splicing mechanisms since various splicing factors were found to be involved at different steps of RdDM pathway [46]. In our study of gene expression changes relative to DMR location, we found two genes adjacent to DMRs that showed increased expression, in accord with the stress-induced hypomethylation of DMRs. On the other hand, the expression of the other genes analyzed was not dramatically changed or even appeared to decrease. In a microarray study in ‘Pokkali’ roots Hruz et al. [47] found that LOC_Os09g15480 (encoding a serine/threonine-rich protein), a gene that we found close to DMR15, is repressed by salt stress.

The identification of DMRs in stress tolerant plants can be a tool to unveil epigenetic regulation of novel salt-responsive genes with putative functional relevance in salt tolerance mechanisms, bringing new clues about how to apply the knowledge of specific methylation variations in stress tolerance management.

## 4. Materials and Methods

### 4.1. Plant Material, Growth Conditions, and Salt Stress Treatment

The salt-tolerant rice variety *Oryza sativa* ssp *indica* cv. Pokkali was used in this study. Seed germination and salt stress imposition followed the procedures described in Ferreira et al. [13]. Briefly, seeds were surface disinfected with a benlate solution (0.1%) for 30 min at 50 °C, rinsed with sterile water, soaked in 70% ethanol for 1 min, and washed with a solution of 2% sodium hypochlorite containing 0.02% Tween 20 for 30 min. After several washes in sterile water, seeds were germinated in Petri dishes containing 3 mm paper embedded in sterile water, in the dark, for 3 days, at 28 °C. Germinated seedlings were transferred to glass tubes containing Yoshida’s medium [48] and allowed to grow in a growth chamber at 28 °C/24 °C and 12 h photoperiod (500 µEm^−2^s^−1^) with 70% humidity. For each condition—salt or control—a pool of 15 seedlings were used. The salt stress treatment was applied to 14-day-old seedlings and consisted in supplementing the Yoshida’s medium with 200 mM NaCl. Rice leaves were collected after 24 h of salt treatment, frozen in liquid nitrogen, and kept at −80 °C.

### 4.2. Methylated DNA Immunoprecipitation Sequencing (MeDIP-Seq)

Genomic DNA from ‘Pokkali’ leaves was isolated using the DNeasy^®^ Plant mini kit (Qiagen, Hilden, Germany) according to the manufacturer instructions. DNA quality was assessed in agarose gel electrophoresis and absorbance spectroscopy using the Nanodrop. DNA was then sonicated to obtain short fragments of approximately 150–400 bp which were then incubated with a monoclonal antibody highly specific to recognize 5-methylcytosine (catalogue n° 39649, Active Motif). The methylated enriched fraction of the immunoprecipitated DNA was high-throughput sequenced using the Illumina Hi-Seq platform as a service provided by Active Motif. The immunoprecipitated DNA was amplified using barcoded Illumina primers to generate the final library for sequencing. A control input library was prepared by amplifying a small amount of DNA (pooling all samples) that did not go through the MeDIP step. Two biological replicates were used per each condition.

### 4.3. Mapping and Processing the MeDIP-Seq Reads

The reference genome sequence and gene annotation information available for Rice (*Oryza sativa* ssp. *japonica*, cv. Nipponbare) is of high quality [49] and the reads were mapped to the Michigan State University Genome Annotation Project Database, version 6.1 (http://rice.plantbiology.msu.edu). To map the raw 50 nt single-end reads, the original reads were computationally processed with the following steps. (1) Quality check of the raw reads using FastQC tool; (2) sequence trimming using Trimmomatic [50], namely the cutting of adapters and other Illumina-specific sequences from the reads, sliding window trimming, standardization to a specified length; (3) mapping and alignment of the processed reads using the program GMAP (Genomic Mapping and Alignment Program) [51]; and (4) duplicate reads were removed with Samtools (public domain: http://samtools.sourceforge.net/ [52]. Only uniquely mapped reads were further analyzed.

### 4.4. Identification of Differentially Methylated Regions (DMRs)

Uniquely mapped reads were analyzed using the MEDIPS software package [53] to estimate methylation levels (using a 20% cut off). The genome was divided into 100 bp windows and each of these was then tested for differential methylation (FDR > 0.1, log2FC(1.2) with minimum mean counts per group = 2. Genomic regions showing statistically significant differential methylation when comparing salt stress versus control conditions were considered as differential methylated regions (DMRs).

### 4.5. Bisulfite Sequencing (BS) of DMRs 

The BS method was used to validate the MeDIP-Seq data by tracking the methylation status of specific identified DMRs. Five hundred nanograms of genomic DNA were subjected to bisulfite conversion using the EZ DNA methylation^TM^ (Zymo Research, Irvine, CA, USA) according to the manufacture’s protocol. Bisulfite-converted DNA (four microliters) was used for PCR amplification of selected regions, namely the DMRs 2 and 15 (see Supplementary Table S1 for primer sequences). The PCR product was cloned into the pCR™4-TOPO^®^ Vector (Invitrogen Life Sciences Technologies, Carlsbad, CA, USA) and used to transform *E. coli* DH5α-competent cells. The plasmidic DNA was extracted and purified with the Easy spin plasmid DNA minipreps kit (Cytomed, Lisbon, Portugal) and about 20 clones were then sent to sequence at Macrogen (http://dna.macrogen.com/eng). The Kismeth platform reported in Gruntman et al. [54] was used to design the primers for amplification of the bisulfite converted DNA and for sequencing analysis of the multiple clones (http://katahdin.mssm.edu/kismeth).

### 4.6. Gene Expression Studies by Real-Time RT-PCR

Total RNA from leaves of ‘Pokkali’ was isolated from a pool of 12 rice seedlings grown for 13 days in control conditions and another 24 h under salt stress (200 mM NaCl). The RNA extraction procedure followed the manufacturer’s instructions (Zymo Resarch). The isolated total RNA was treated with TURBO DNA-free (Ambion, Invitrogen) to eliminate any possible DNA trace. The RNA integrity was checked by agarose gel electrophoresis and RNA concentration and purity was measured with Nanodrop. The cDNA synthesis was performed with 4 µg of total RNA using the Random Hexamer primer and according to the instructions of the Transcriptor High Fidelity cDNA Synthesis Kit (Roche, Basel, Switzerland). The total cDNA obtained was diluted 5 times and 5 µL were used for PCR amplification. Real-time quantitative PCR was performed using the LightCycler 480 system (Roche) and the SYBR Green I Master mix (Roche). PCR running conditions: one cycle at 95 °C for 5 min and 45 cycles at 95 °C for 10 s, 60 °C for 10 s and 72 °C for 10 s. The *C*t values were calculated from means of three technical PCR replicates. The relative expression level of each transcript was calculated using the method “relative quantification with kinetic PCR efficiency correction”. The rice gene ubiquitin-conjugating enzyme E2 (*OsUBC2,* LOC_Os02g42314) was used to normalize the relative expression of the target transcripts given our previous experiments showing its stability under salt stress as referred in Ferreira et al. [13]. All experiments were done with at least three biological replicates. Primers for genes located nearby selected DMRs are listed in Supplementary Table S2.

## Figures and Tables

**Table 1:** Summary of methylated DNA immunoprecipitation (MeDIP-seq) data analysis. The input refers to a control library that did not go through the MeDIP procedure.

Condition	Biological Replicates	Total Reads	# Uniquely Mapped Reads	% Uniquely Mapped Reads	Cytosine Coverage % (Total C’s = 63095915)						
					0×	1×	2×	3×	4×	5×	>5×
Control 1	2	17.225.011	4.283.278	24.87	82.82	8.05	2.48	1.37	0.92	0.67	3.7
Control 2	2	16.081.432	4.075.168	25.34	84.54	6.63	2.32	1.34	0.91	0.66	3.59
Stress 1	2	13.681.641	3.639.466	26.60	82.86	8.89	2.46	1.29	0.84	0.61	3.05
Stress 2	2	13.845.643	3.562.794	25.73	84.54	6.63	2.32	1.34	0.91	0.66	3.59
Input	1	14.661.478	7.016.939	47.86	51.2	22.17	14.59	7.39	3.03	1.06	0.57

**Table 2:** List of DMRs between control and salt stress imposed on 14 days-old rice seedlings. The position of DMRs at chromosome level is schematically represented. Genes nearby DMRs are indicated.

Chr	DMR ID	Coordinat. Start End	Repeat Masker Annotation	Gene Annotation	DMR Position Relative to the Gene	Gene Description
I 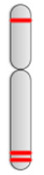	1	3431001 3431100	AnacC1 transposon (ORSiTETNOOT00122)	LOC_Os01g07270	78 bp downstream	Transposon
				LOC_Os01g07280	506 bp downstream	Disease-resistance protein
	2	38016401 38016700	-	LOC_Os01g65490	2100 bp upstream	DNA binding protein
				LOC_Os01g65500	750 bp upstream	Chloride channel protein
	3	39466301 39466500	(CGG)n rich area	LOC_Os01g67910	5’ overlap	Expressed protein
				LOC_Os01g67920	796 bp downstream	Tetratricopeptide repeat protein
II 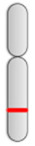	4	26500001 26500100	-	LOC_Os02g43890	Within (intron/exon/intron)	Hypothetical protein
III 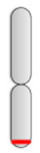	5	36070201 36071200	AnacA2 transposon (ORSiTETNOOT00130)	LOC_Os03g63840	4194 bp downstream	Expressed protein
				LOC_Os03g63850	1972 bp upstream	OsFBDUF19 protein
IV 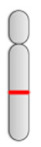	6	22831201 22831400	(CGG)n rich area	LOC_Os04g38390	780 bp downstream	Wound/stress protein
				LOC_Os04g38400	2620 bp upstream	Ethylene-insensitive 3 protein
V 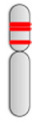	7	4804401 4804700	AnacA10 transposon (ORSiTETNOOT00124)	LOC_Os05g08760	Within (exon/intron)	Expressed protein
	8	4805301 4805500	-	LOC_Os05g08760	Within (exon)	Expressed protein
	9	9320201 9320400	RIRE3 gypsy-type retrotransposon (ORSiTERTOOT00027)	LOC_Os05g16420	1570 bp downstream	SHR5-receptor-like kinase protein
				LOC_Os05g16430	1300 bp upstream	SHR5-receptor-like kinase protein
VI 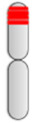	10	962901 963200	E4 repeat sequence (ORSiOTOT00000050)	LOC_Os06g02680	680 bp upstream	Expressed protein
				LOC_Os06g02690	20 bp downstream	Expressed protein
	11	970501 970600	-	LOC_Os06g02700	Within (exon)	Retrotransposon Ty3-gypsy
	12	983401 983500	-	LOC_Os06g02730	3591 bp upstream	Aspartic proteinase nepenthesin-2 precursor protein
				LOC_Os06g02740	7261 bp upstream	Retrotransposon
	13	1010401 1010700	(CGG)n rich area	LOC_Os06g02770	Within (exon)	Expressed gene
VIII 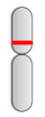	14	9021501 9021600	-	LOC_Os08g14950	1150 bp downstream	Receptor-like kinase 2 precursor protein
				LOC_Os08g14960	4240 bp upstream	Receptor-like kinase precursor protein
IX 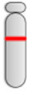	15	9475001 9475300	Ty3-gypsy retrotransposon (ORSiTERT00200079)	LOC_Os09g15470	3500 bp upstream	Retrotransposon Ty3-gypsy
				LOC_Os09g15480	1100bp downstream	Ser/Thr-rich protein
XI 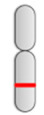	16	20435601 20436000	-	LOC_Os11g34870	Within (intron)	Expressed protein
XII 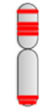	17	1446901 1447100	AnacA10 transposon (ORSiTETNOOT00124)	LOC_Os12g03601	519 bp upstream	Expressed protein
				LOC_Os12g03610	2283 bp upstream	Expressed protein
	18	4989301 4989600	noaCRR2 retrotransposon (ORSiTERTOOT00141)	LOC_Os12g09500	975 bp upstream	Cytochrome P450 protein
				LOC_Os12g09510	8570 bp upstream	Hypothetical protein
	19	5108601 5108800	Ty3-gypsy retrotransposon (ORSiTERT00200079)	LOC_Os12g09680	Within (intron)	Retrotransposon Ty3-gypsy
	20	5301501 5301700	Centromere-like LTR transposon (ORSiCMCM00100011)	LOC_Os12g10000	2500 bp upstream	Retrotransposon
				LOC_Os12g10010	34 bp downstream	Expressed protein
	21	25340601 25341000	(GGA)n rich area	LOC_Os12g40930	155 bp upstream	Expressed protein
				LOC_Os12g40940	4377 bp upstream	Expressed protein
	22	25763601 2764100	noaCRR2 retrotransposon (ORSiTERTOOT00141)	LOC_Os12g41630	4000 bp upstream	OsFBX463–F-box domain protein
				LOC_Os12g41634	Within (exon)	Expressed protein
				LOC_Os12g41640	800 bp upstream	Expressed protein

## Supplementary Materials

The following are available online at http://www.mdpi.com/2075-4655/3/1/4/s1, Figure S1: Chromosome-level view of DNA methylation in control (A) and salt stress (B) conditions. The chromosome read coverage plots were based on the average RPM over 100,000 bp windows. Figure S2: Methylation status of all DMRs identified between control and salt stress conditions. Figure S3: Bisulfite sequencing (BS) analysis for DMR2 and DMR15. The region analyzed by BS is indicated with a grey line under the DMR (black box). Positions of the first and last cytosines analyzed are indicated. Cytosine methylation contexts are symbolized by red circles for CG, green circles for CHG and blue circles for CHH (H=A, T or C). Methylation (at the different contexts and globally) is also indicated. Figure S4: Gene Ontology (GO) analysis. Multilevel pie chart representation of GO annotations for genes near salt stress-induced DMRs. Charts were built using Blast2GO and are represented according to (A) molecular function (B) cellular component (C) biological process. Table S1: List of primers used for Bisulfite Sequencing (BS)-PCR analysis of specific DMRs. For PCR amplification of DMRs, the Taq DNA polymerase (New England Biolabs) was used. Table S2: List of primers used for expression analyses of rice genes located nearby DMRs. Details on real-time RT-PCR conditions are described in material and methods.
