# Supplementary material for: Uncovering Differentially Methylated Regions (DMRs) in a Salt-Tolerant Rice Variety under Stress: One Step towards New Regulatory Regions for Enhanced Salt Tolerance
Source: Epigenomes. 2019 Jan 18;3(1):4. doi: 10.3390/epigenomes3010004 (PMC8594724; doi:10.3390/epigenomes3010004)
Supplement: Supplementary file 1 [file epigenomes-03-00004-s001.pdf]

SUPPLEMENTARY INFORMATION

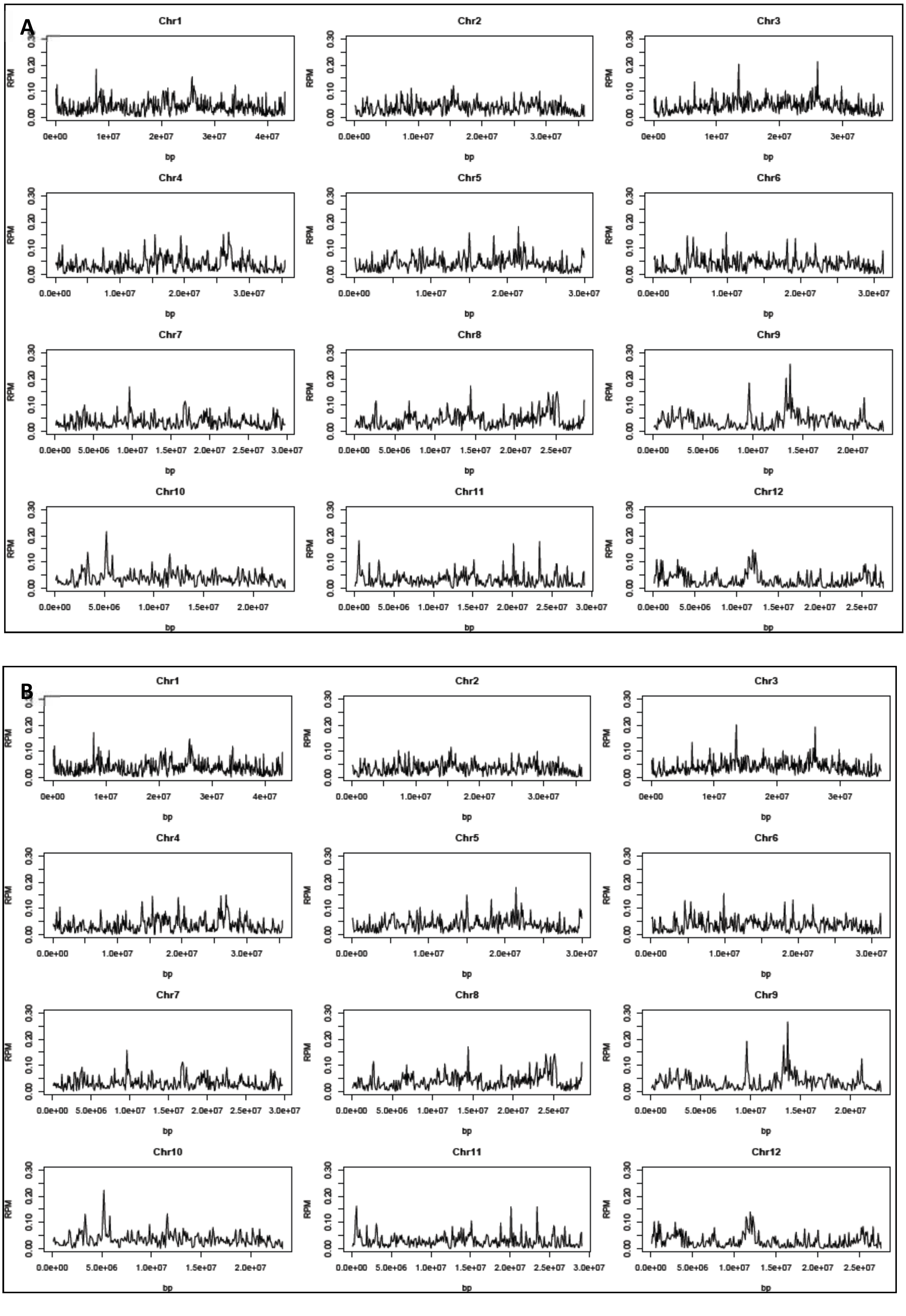

Figure S1- Chromosome-level view of DNA methylation in control (A) and salt stress (B) conditions. The chromosome read coverage plots were based on the average RPM over 100,000 bp windows.

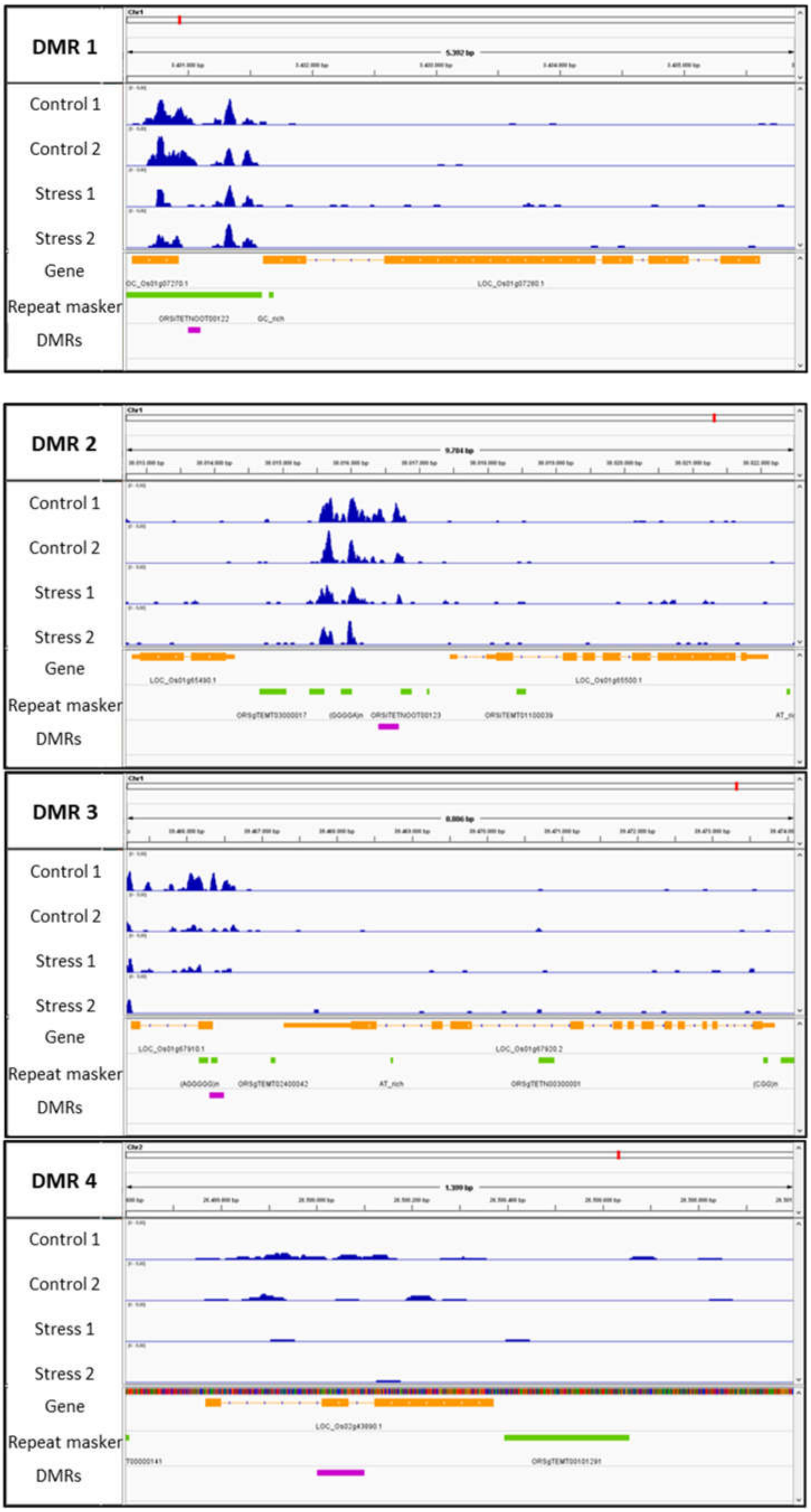
DMR 1
Control 1
Control 2
Stress 1
Stress 2
Gene
Repeat masker
DMRs
DMR 2
Control 1
Control 2
Stress 1
Stress 2
Gene
Repeat masker
DMRs
DMR 3
Control 1
Control 2
Stress 1
Stress 2
Gene
Repeat masker
DMRs
DMR 4
Control 1
Control 2
Stress 1
Stress 2
Gene
Repeat masker
DMRs

Figure S2

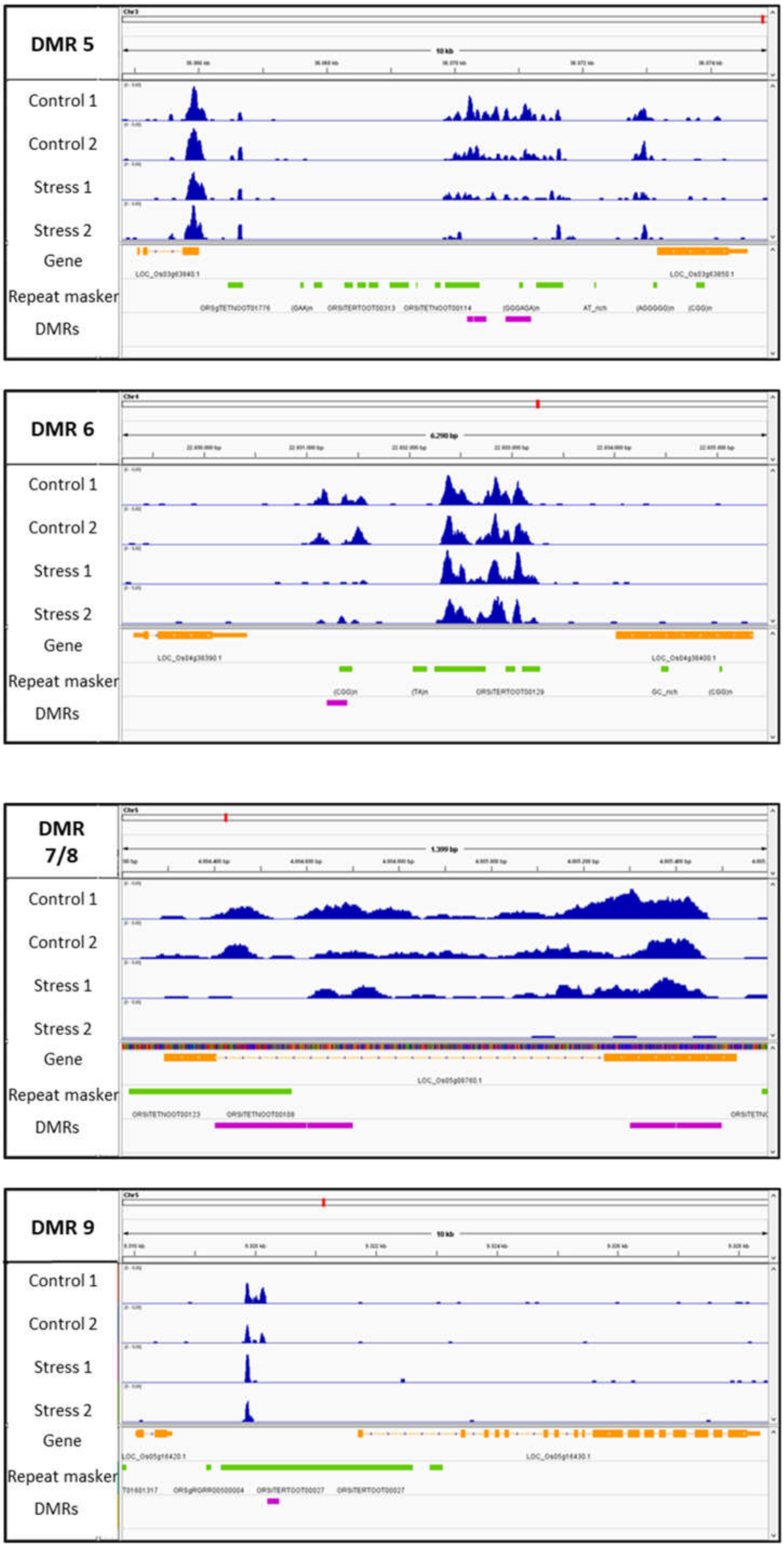

Figure S2 (Cont.)

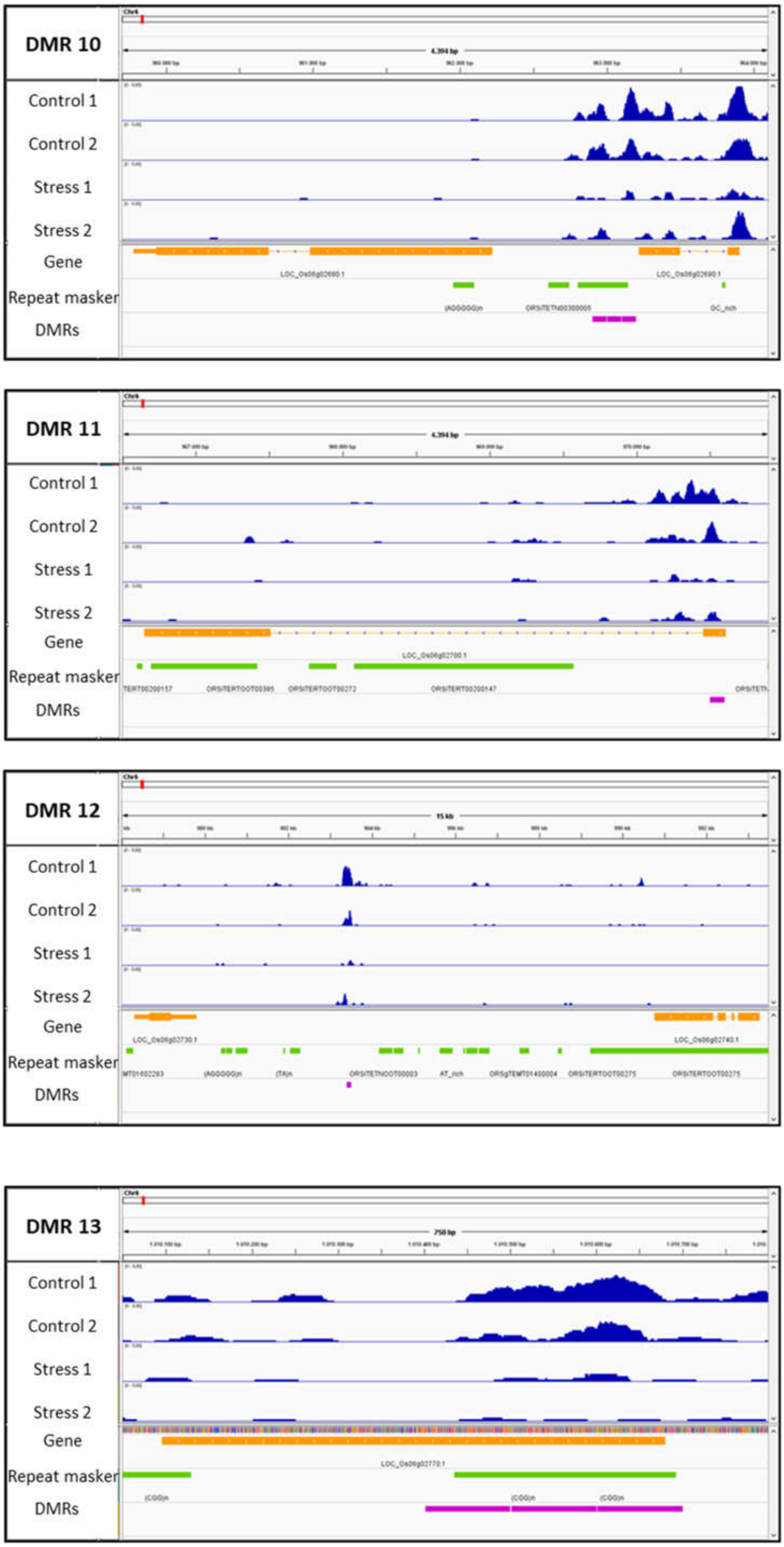

Figure S2 (Cont.)

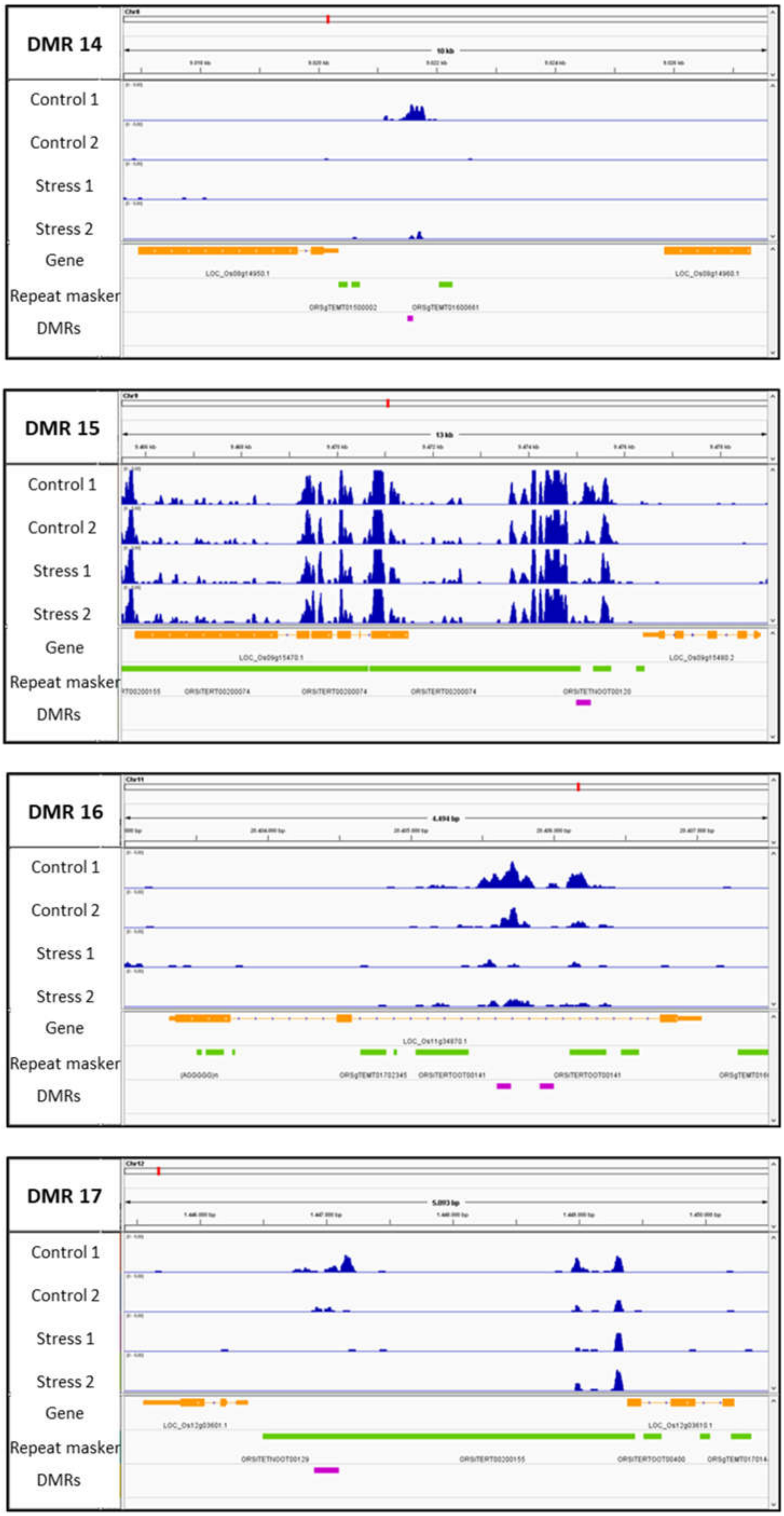

Figure S2 (Cont.)

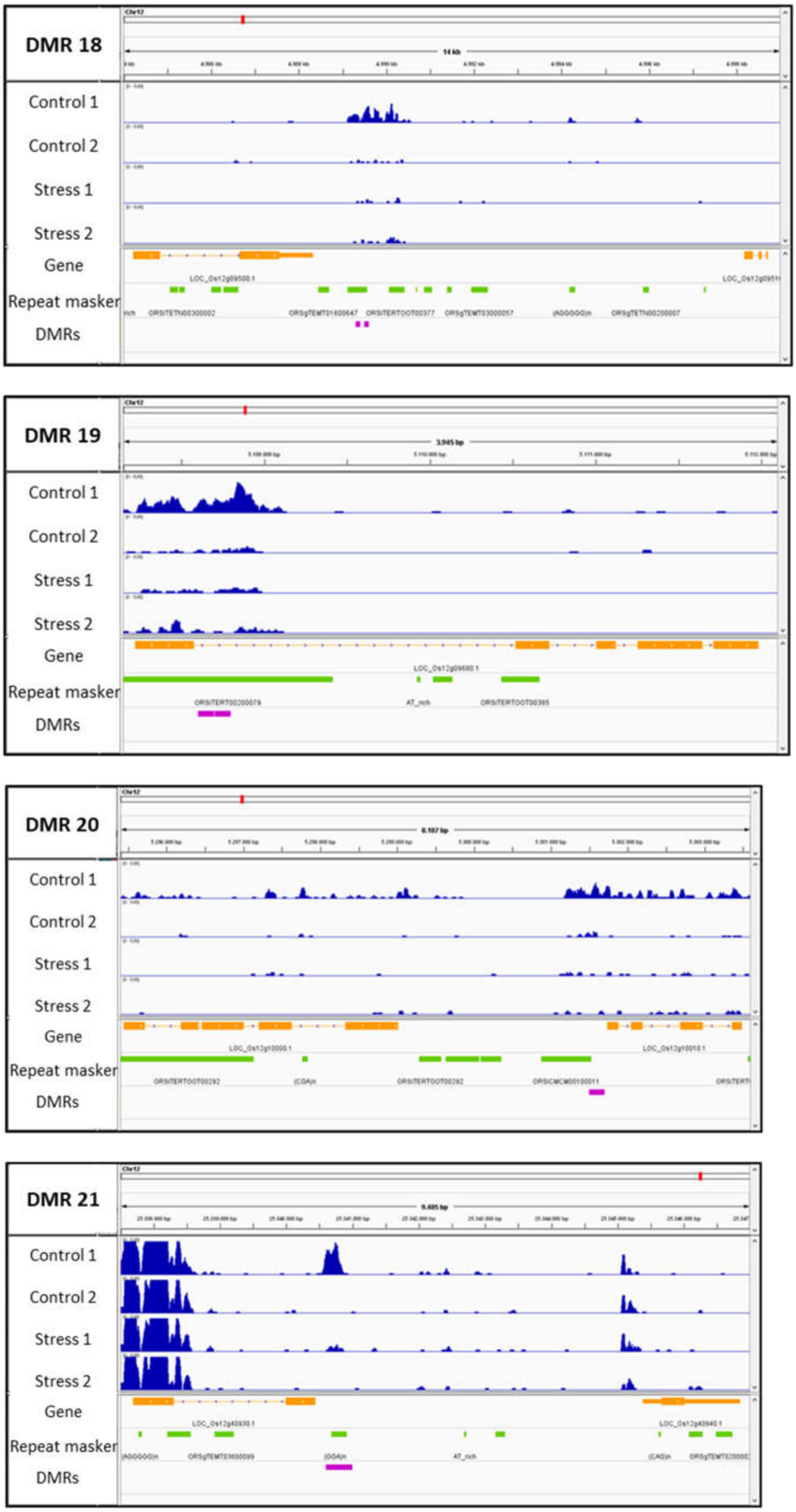

Figure S2 (Cont.)

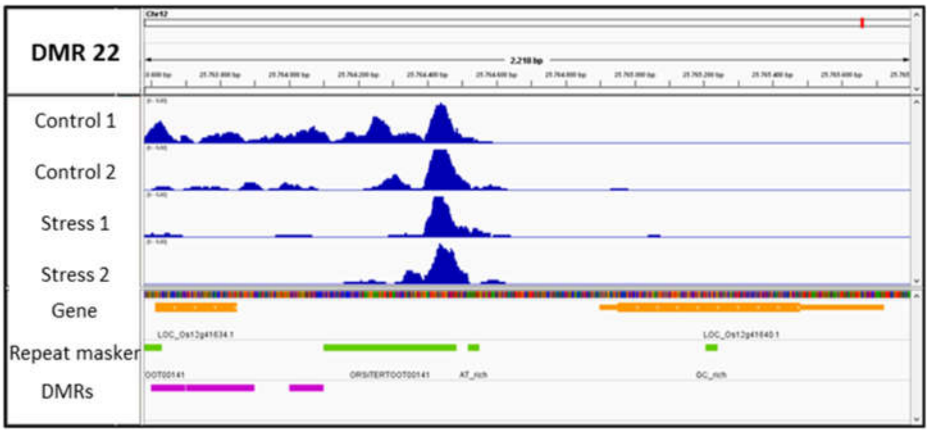

Figure S2- Methylation status of DMRs identified between control and salt stress conditions.

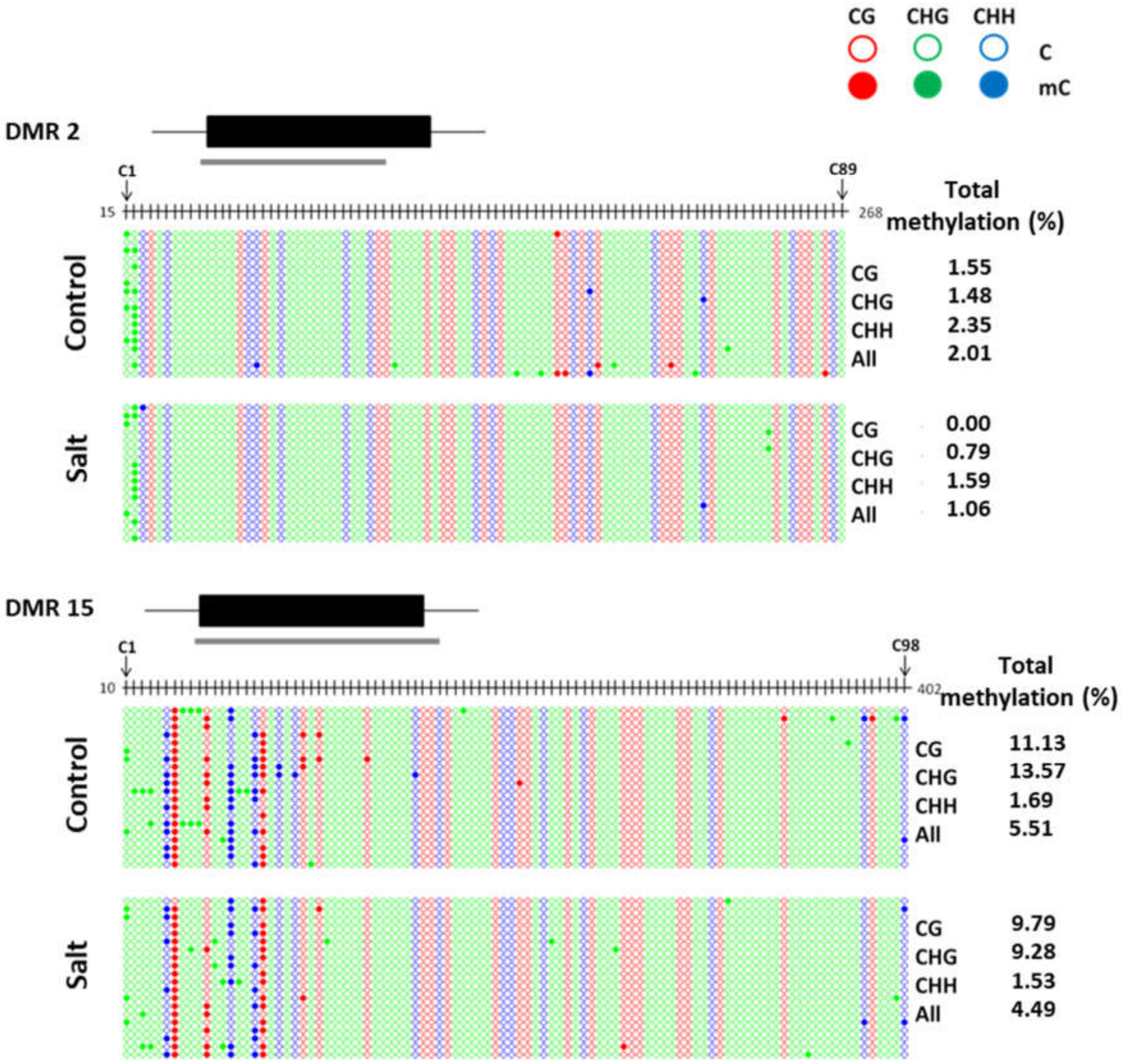

Figure S3- Bisulfite sequencing (BS) analysis for DMR2 and DMR15. The region analysed by BS is indicated with a grey line under the DMR (black box). Positions of the first and last cytosines analysed are indicated. Cytosine methylation contexts are symbolized by red circles for CG, green circles for CHG and blue circles for CHH (H=A, T or C). Methylation (at the different contexts and globally) is also indicated.

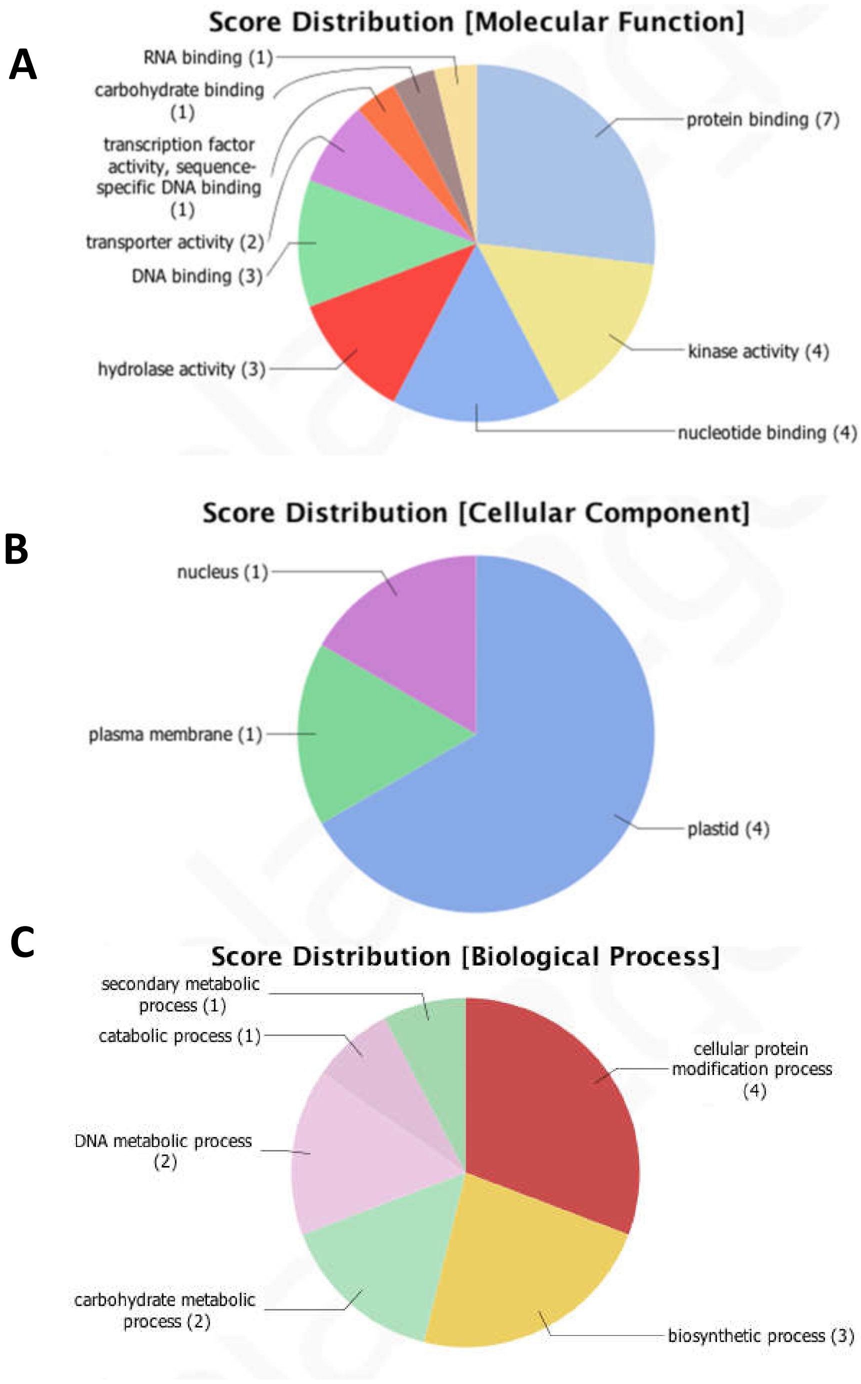

Figure S4- Gene Ontology (GO) analysis. Multilevel pie chart representation of GO annotations for genes in close proximity to salt stress-induced DMRs. Charts were built using Blast2GO and are represented according to (A) molecular function (B) cellular component (C) biological process.

Table S1- List of primers used for Bisulfite Sequencing (BS)-PCR analysis of specific DMRs. For PCR amplification of DMRs, it was used the Taq DNA polymerase (New England Biolabs).

DMR	Forward Primer	Reverse Primer
DMR 2	GTGGATGATGGGTGYYAAAGTAGGA	TCCTCTTCTCTCCTCTTCCATTA
DMR 15	TGTTATTTGYATTGATTTAATAGGG	ACTACCTCCCTCTCTCTCTCA

Table S2- List of primers used for gene expression analyses based on DMR location. Details on real-time RT-PCR conditions are described in material and methods.

Genes	Forward Primer	Reverse Primer
LOC_Os01g65490	TTGTACATGCAGGGCACCTC	GTACGTCGACTGGTCCAGAG
LOC_Os01g65500	GCGCTTCTCTGGAGGACATT	CGCTGCGGCAGTACTCTATT
LOC_Os02g43890	TGTCACAGACCGCTACATCC	GAAGAATGCACCCACTCCGT
LOC_Os05g16430	AGAGCCTACAAACCTTGGGG	TTCCATTTGAGGCCAGGGAA
LOC_Os06g02700	TGATGAAGTTCTTCTGGCCG	ACAACAATATCGGCTCGGGG
LOC_Os06g02770	GATTTGGGTGTGGCCGAGTG	CACCAAGCAGCGAGCAAC
